# Telehealth based parental support over 6 months improves physical activity and sleep quality in children with autism: a randomized controlled trial

**DOI:** 10.3389/fped.2024.1496827

**Published:** 2024-10-25

**Authors:** Xin Shen, Peiying Huang, Qian Liu, Yin Guo, Lan Zheng

**Affiliations:** ^1^ Key Laboratory of Physical Fitness and Exercise Rehabilitation of Hunan Province, Hunan Normal University, Changsha, China; ^2^ College of Physical Education, Hunan Normal University, Changsha, China

**Keywords:** exercise, health behavior, insomnia, sleep disturbance, telemedicine

## Abstract

**Purpose:**

Sleep disturbances are prevalent in autistic children. The emergence of telehealth offers new possibilities for remote professional intervention. By combining telehealth with parental support, this study aims to explore a novel family-based model to enhance moderate-to-vigorous physical activity (MVPA) and improve sleep quality in children with autism.

**Methods:**

Thirty-four autistic children (mean age = 15.7 years) were randomly assigned to either a 6-month intervention group or a control group. Both groups received standard physical education classes at school. The intervention group received additional after-school telehealth support. MVPA and sleep quality were assessed 1 week before the intervention and at the 6-month follow-up.

**Results:**

After 6 months, children in the intervention group nearly doubled their daily MVPA compared to the control group (Cohen's *d* = 8.34, CI_95%_ = 6.17–10.52). Actigraphy-assessed sleep efficiency was notably higher (*d* = 2.35, CI_95%_ = 1.44–3.26), and there were reductions in wake time (*d* = 1.65, CI_95%_ = 0.84–2.46), sleep fragmentation (*d* = 0.80, CI_95%_ = 0.07–1.52), and sleep latency (*d* = 0.82, CI_95%_ = 0.09–1.54) were all reduced. These improvements in objective sleep metrics were corroborated by subjective assessments using the Sleep Disturbance Scale for Children (*d* = 0.86, CI_95%_ = 0.13–1.59).

**Conclusions:**

Telehealth combined with parental support addresses barriers to enhancing health behaviors at home. This innovative model not only improves after-school MVPA and sleep quality in autistic children but also holds significant potential for benefiting other populations requiring remote support.

**Clinical Trial Registration:**

https://clinicaltrials.gov/study/NCT06444659?id=NCT06444659&rank=1 (NCT06444659).

## 1 Introduction

Autism spectrum disorder (hereafter referred to as autism), is characterized by persistent deficits in social communication and interaction, along with restricted and repetitive patterns of behavior, with a global prevalence of approximately 1% (1). Sleep disorders are a common comorbidity in children with autism (2). Issues such as insomnia, irregular sleep-wake cycles, and frequent nocturnal awakenings exacerbate the medical, psychiatric, and psychosocial challenges associated with autism, placing a significant burden on families. Sleep interventions typically involve pharmacological, behavioral, or combined approaches. While medications such as melatonin supplementation are widely used, their long-term efficacy is variable (3, 4). Behavioral interventions often require professional involvement (5), which can limit accessibility. On the other hand, a substantial body of research supports the benefits of physical activity, particularly moderate-to-vigorous physical activity (MVPA), in improving sleep quality in autistic children (6). Given the limitations of mainstream interventions, engaging in physical activity presents an effective and safer alternative.

Promoting physical activity in children requires basic executive functions and attention, areas in which autistic children often struggle (7, 8). This unique condition necessitates more structured interventions, with a strong emphasis on motivational support. Most previous studies have utilized school-based exercise interventions to promote physical activity in autistic children. For instance, in one study, autistic children participated in a 12-week basketball skills program, and post-intervention assessments showed significant improvements in sleep quality during weekdays (9). However, addressing these clinical conditions requires more than just school-based environments. Developing health behaviors also depends on consistent parental support and a conducive after-school environment. Parents, driven by their commitment to their children's well-being, are uniquely positioned to provide the necessary daily support, promoting sustained engagement in positive health behaviors. Despite this, research on the impact of family support on physical activity and subsequent improvements in sleep quality for autistic children remains scarce.

Extensive research has established the critical role of parental involvement in improving sleep quality in children (10). The biopsychosocial model of sleep highlights that the balance between sleep and wakefulness is sensitive to perceived environmental and social threats, which can heighten arousal and awareness, negatively impacting sleep quality (11). For autistic children, their sense of safety is especially influenced by their social and emotional environment. Parental support provides a sense of physical and emotional security, which may be crucial for promoting daily physical activity and good sleep hygiene. While previous research has demonstrated the essential role of parents in either promoting physical activity or improving sleep quality among autistic children, it remains unclear whether parental support in a home environment can effectively improve both physical activity and sleep quality in this cohort. This gap suggests that current exercise interventions have not fully addressed the influence of family factors. From a practical standpoint, daily home-based interventions are just as important as school-based programs for fostering healthy behaviors.

Additionally, parents may generally lack the specialized knowledge needed to understand the unique demands and strategies required for behavioral modifications in autistic children, which often necessitates professional guidance. With advancements in technology, remote video consultations with experts have become possible for many services that traditionally required on-site visits (12). Telehealth refers to healthcare services provided through digital communication technologies (13). This modality has rapidly evolved over the past few decades, particularly during the coronavirus disease 2019 (COVID-19) pandemic, emerging as an effective and cost-efficient means of delivering and accessing high-quality healthcare services. Although this approach has not yet been widely adopted, early findings suggest it has a promising effect on promoting physical activity (14). To our knowledge, no study has assessed the effectiveness of telehealth in addressing the health needs of individuals with autism. Therefore, the purpose of this study was to explore a novel telehealth-based parental support model to evaluate its effectiveness in improving MVPA and sleep quality in autistic children.

## 2 Methods

### 2.1 Study design

This randomized controlled trial (clinical registration: NCT06444659) was approved by the Ethics Committee of Hunan Normal University (Approval No. 301 08/05/2023) and conducted in accordance with the Declaration of Helsinki. Two groups of families were recruited to voluntarily participate in either a 6-month intervention or control period. While the primary outcome of this study is sleep metrics, the power analysis was based on MVPA due to its strong correlation with sleep hygiene, as an improvement in MVPA is expected to lead to better sleep quality. Assuming a *p*-value of 0.05 and a power of 80% in a two-tailed test, the minimum number of participants required to demonstrate statistical significance for this pre-post, two-arm design is 15 per group, with a Cohen's *d* of 1.1. Considering a potential dropout rate of 10%, a total of 34 participants were required.

### 2.2 Participants

Potential families were contacted through a local special education school in Changsha, Hunan Province, and informed about the study's aims, potential benefits, and risks. To participate, families had to meet a set of inclusion and exclusion criteria. The inclusion criteria were: children had a confirmed diagnosis of autism as per the Diagnostic and Statistical Manual of Mental Disorders, 5th Edition; children were between 12 and 15 years old; they had no contraindications to physical activity; and they had not regularly engaged in structured exercise beyond school-based physical education classes for at least 3 months prior to the study. The exclusion criteria were: children with other neurodevelopmental or psychiatric disorders; any hearing, vision, or physical impairments; any severe clinical symptoms that could interfere with participation; a history of psychotropic medication use that could influence sleep within the past 3 months; the use of sleep medications within the past 3 months or during the course of the study; and missing more than two regular school-based physical education classes or telehealth sessions. A total of 34 families participated in the study, and written informed consent was provided by the children's legal guardians. Additionally, we utilized the Childhood Autism Rating Scale (CARS) to assess the severity of autism symptoms in each participant, ensuring that variations in severity did not confound the study's results. The CARS consists of 15 items, each addressing a different aspect of autism. Each item is rated on a scale from 1 to 4, with total scores ranging from 30 to 37 indicating mild to moderate autism, while scores above 37 denote severe autism (15). The demographics of the participating children are presented in Table 1.

**Table 1:** Demographics.

Variable	Control group	Intervention group
*N* of boys/girls	9/8	10/7
Age (years)	15.9 ± 0.2	15.5 ± 0.2
Height (cm)	164.3 ± 9.0	163.2 ± 7.8
Weight (kg)	62.2 ± 9.2	60.8 ± 5.1
BMI (kg/m^2^)	23.1 ± 2.4	22.9 ± 1.5
CARS	33.0 ± 2.6	32.8 ± 2.6

Data are expressed as mean ± standard deviations. CARS, childhood autism rating scale.

### 2.3 Telehealth protocol

Both the intervention and control groups participated in the standard three-times-per-week physical education classes at school. After school, participants in the control group received no additional support from the research team, while those in the intervention group received regular telehealth support as follows:

The expert-family telehealth protocol was delivered through Tencent's VooV Meeting platform, with three 30-minute sessions held each week. The expert team consisted of a certified autism specialist and an exercise scientist with expertise in pediatric physical activity. The live-conference training sessions included guidance on establishing a supportive home environment, managing anxiety, implementing structured relaxation routines—such as yoga and mindfulness practices—and creating stable evening routines with positive reinforcement to motivate the child to engage in physical activities. In addition, parents utilized video check-ins to ensure proper implementation of personalized routines and to make adjustments as needed. This regular interaction allowed for real-time feedback and tailored modifications to meet each child's individual needs.

### 2.4 Evaluations

Children's physical activity and sleep metrics were assessed 1 week before the study and 1 week after the 6-month period. The ActiGraph GT3X+ (ActiGraph LLC, USA) was used to measure physical activity levels and sleep patterns (16, 17). Children wore the accelerometer on their right hip continuously for 7 days, including 5 weekdays and 2 weekend days, removing it only during bathing. Researchers contacted parents daily to ensure adherence and monitor device usage. The collected data included time spent in MVPA and sleep metrics. Additionally, the Sleep Disturbance Scale for Children, suitable for children aged 6–16 years (18), was used to subjectively assess the severity of sleep disturbances. The scale comprises 26 items, each rated on a 5-point Likert scale, with the total score calculated by summing the scores across all dimensions. A total score above 39 indicates the presence of sleep disturbance (19).

### 2.5 Statistics

All statistical analyses were conducted using R version 4.4.1 (Race for Your Life). Data structure was initially verified using the Shapiro-Wilk test for normality and the Levene test for homogeneity of variances. For non-Gaussian data, a natural logarithm transformation was applied, and preliminary tests for statistical assumptions were repeated. A mixed analysis of variance was used to compare the effects of time and treatment. When significant interactions were observed, simple main effects (group) were examined, and follow-up pairwise comparisons were conducted using the Holm method. Pearson's chi-square test was used to compare overall rates of subjective sleep disturbance between groups. An alpha level of 0.05 was set for statistical significance.

## 3 Results

**Figure 1:**
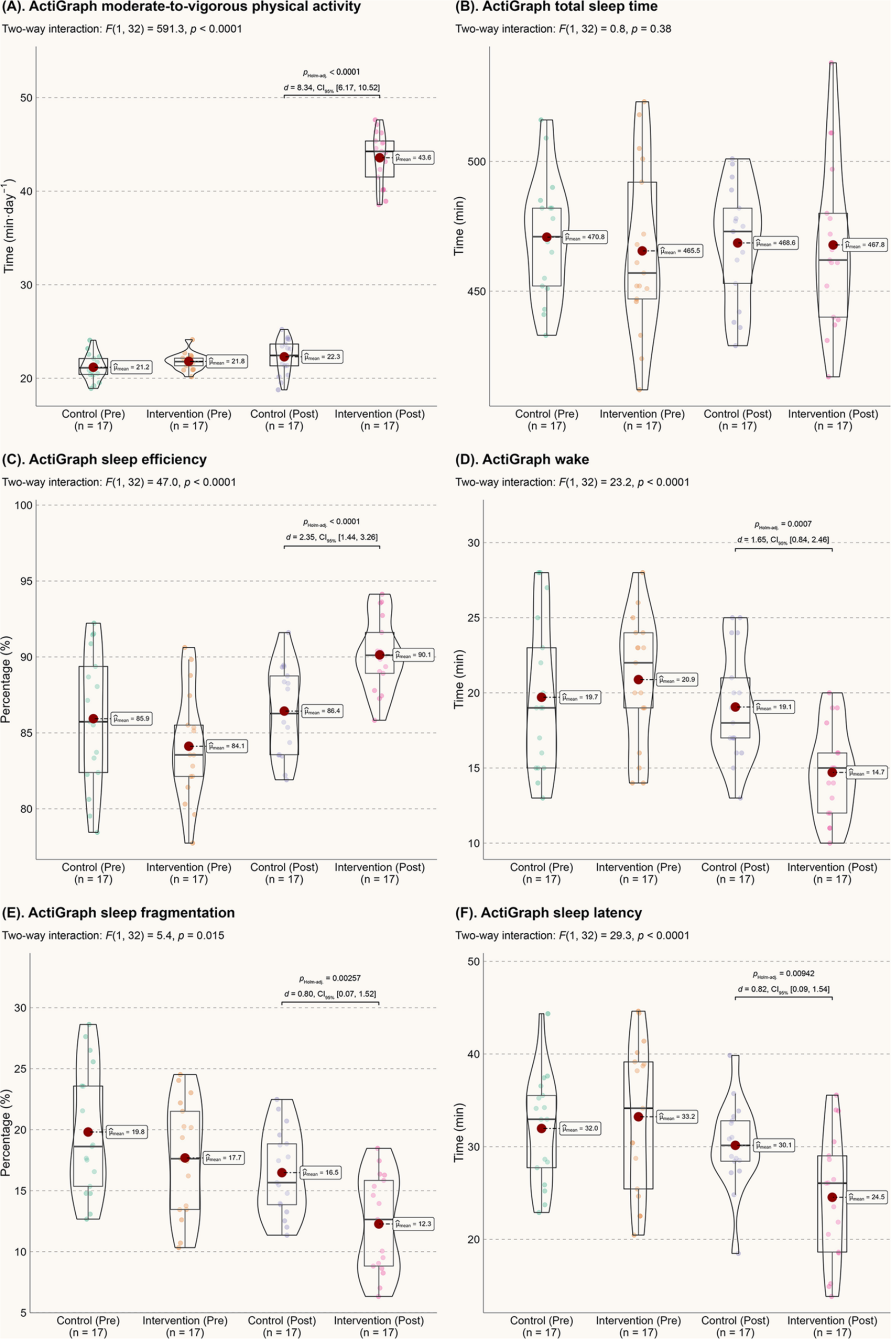
Actigraph data. **(A)** MVPA; **(B)** Total sleep time; **(C)** Sleep efficiency; **(D)** Wake time after sleep onset; **(E)** Sleep fragmentation index; **(F)** Sleep onset latency.

Figure 1 presents the ActiGraph data. After 6 months, children in the intervention group nearly doubled their daily MVPA time (mean of 43.6 min for the intervention group vs. 22.3 min for the control group). Although there was no significant difference in total sleep time, the intervention group showed significant improvements in objective sleep metrics. Compared to the control group, the intervention group had an average increase in sleep efficiency by 3.7 percentage points, a reduction in wake time by 4.4 min, a decrease in sleep fragmentation by 4.2 percentage points, and a reduction in sleep latency by 5.6 min (all *p*-values less than 0.01).

**Figure 2:**
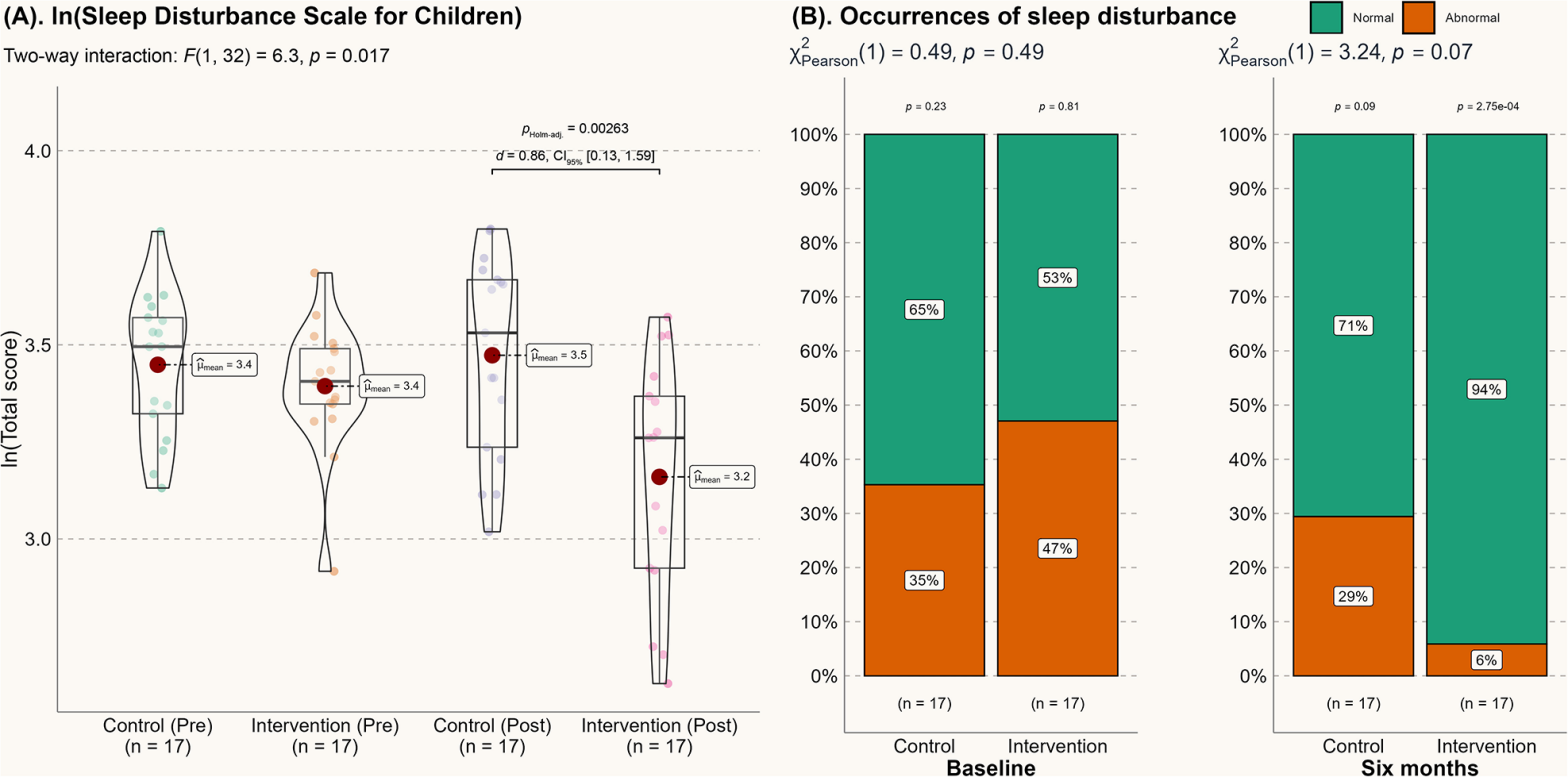
Score from the sleep disturbance scale for children. **(A)** Score from the sleep disturbance scale for children; **(B)** Changes in the incidence of abnormal sleep disturbances.

These improvements in sleep quality are further supported by subjective sleep assessments. As shown in Figure 2A, the log-transformed total score for sleep disturbance was significantly reduced at the 6-month mark (*p* < 0.01). Although the between-group comparison did not reach statistical significance at the five percent level (*p* = 0.07, as shown in Figure 2B), the number of children with abnormal sleep disturbance in the intervention group was significantly reduced after 6 months (*p* = 0.00651). Thus, we conclude that the reduction in abnormal sleep disturbances over time is likely attributable to the intervention.

## 4 Discussion

It is well established that parental support plays a crucial role in developing health behaviors in autistic children. This study establishes key connections between expert-guided telehealth platforms and their effectiveness in enhancing MVPA and improving various sleep metrics in autistic children, thereby contributing to the literature. Our method uniquely combines telehealth with a structured physical activity program, allowing for real-time feedback and personalized support tailored to each child's unique needs. This integration not only empowers parents to actively participate in their child's health behaviors but also addresses barriers that traditional school-based approaches may overlook. Compared to broader school-based approaches, this model offers a more comprehensive strategy for sustaining behavior change. Consequently, we provide the first empirical evidence supporting the use of telehealth to improve the quality of life for children with autism.

Regular physical activity, particularly moderate-to-vigorous physical activity (MVPA), offers various health benefits throughout life. However, autistic children often exhibit different behavior patterns from their typically developing peers due to poor executive functions and attention deficits (7, 20), which can hinder their engagement in MVPA. Previous studies have successfully used school-based exercise interventions to improve gross motor skills and physical activity in autistic children (21, 22). However, research on after-school physical activity for autistic children is limited, and a major barrier to participation is a lack of time among parents (23), highlighting a family-related opportunity to enhance MVPA. Healy and colleagues, through interviews with 13 families of autistic children, suggested that reminding parents of the importance of physical activity is essential for motivating both themselves and their children to engage in it (24). Our results provide empirical evidence supporting this theory, demonstrating that planned telehealth video consultations and follow-ups can actively motivate parents to encourage their children's overall leisure-time physical activity, thereby improving daily MVPA. This underscores the critical role of parental engagement in fostering proactive living habits within this cohort.

Despite the substantial impact of telehealth-based parental support on physical activity, the participants’ daily MVPA only increased to 44 min, which is below the World Health Organization's recommended 60 min of daily physical activity for this age group (25). This study did not impose a mandatory daily physical activity requirement on participating families, considering the diverse conditions of each family. While future studies could explore setting specific exercise goals, the primary focus should remain on fostering lasting behavioral changes for both parents and their children. Over the 6-month period of continuous professional support, the aim was for parents to integrate proactive living into their routine family educational philosophy and, in turn, enhance their children's positive attitudes towards exercise and sports during this critical developmental stage. In the long term, it is hoped that autistic individuals will voluntarily engage in physical activity throughout their lives, thereby benefiting from improved physical and mental health due to their proactive living habits. A longer follow-up study is needed to validate this hypothesis.

Autistic children with sleep problems may be particularly vulnerable to irritability, inattentiveness, or motor deficiencies, which can exacerbate their clinical challenges. Therefore, the improvement in various sleep metrics observed in this study is highly significant for these children and their families. Both groups in this study achieved approximately 8 h of daily sleep (Figure 1A), meeting the lower end of the recommended sleep duration (26). However, for individuals with sleep issues, sleep quality is often a more reliable indicator of overall sleep experience than sleep quantity (27). After the 6-month intervention, autistic children showed significant improvements in objective sleep efficiency (*d* = 2.35), reduced wake time (*d* = 1.65), decreased sleep fragmentation (*d* = 0.80), reduced sleep latency (*d* = 0.82), and subjective sleep disturbance (*d* = 0.86). From a probabilistic perspective (28), these improvements reflect a 71.4%–92.1% probability of superiority in sleep quality compared to the control group. Notably, these effects appear to surpass those of melatonin treatment (29) and behavioral sleep interventions (30) for autism. From both economic and treatment effectiveness standpoints, the current home-based, parental support-induced physical activity model is preferable for enhancing the sleep quality of autistic children.

The improved sleep quality observed in our sample may be attributed to three main factors. First, increased Physical Activity: Improvement in daily MVPA is associated with better sleep quality. Existing evidence generally supports the relationship between increased physical activity and improved nocturnal sleep health among children and adolescents (31). The elevated melatonin levels following exercise have been proposed as a potential mechanism (32), though further research is needed to confirm these findings. Additionally, improved sleep quality can enhance daytime physical activity (33), creating a positive feedback loop that promotes overall physical and mental health. Second, the telehealth meetings introduced anxiety management strategies, which may have helped parents create a more supportive and relaxed home environment. Strong parent-child relationships provide sustained support for long-term health behavior changes. A coherent family environment is associated with longer sleep duration, reduced nighttime awakenings, and more consistent sleep patterns in children (34, 35). Through expert consultation and feedback, parents became more effective at offering autonomy support, establishing clear behavioral expectations, and providing consistent emotional reinforcement. This approach fostered a secure and inclusive environment for autistic children and effectively reduced psychological barriers to physical activity. Third, this study addresses a gap in research on after-school interventions for sleep quality in autistic children. There may be a causal relationship between night-time physical activity and improved sleep quality. While some theories suggest that evening exercise could blunt nocturnal melatonin increases (36, 37), experimental studies challenge this link (38, 39) and suggest other factors at play. For example, acute bouts of exercise can elevate gamma-aminobutyric acid (GABA) levels in the cortex (40, 41), and GABA's brain relaxant effects are well established (42). We hypothesize that through parental support, autistic children began engaging in regular after-school and even night-time physical activities, leading to elevated GABA levels and subsequently better sleep quality. This hypothesis opens new avenues for future research. We recommend exploring different exercise modalities and intensities to further investigate their impact on GABA levels and sleep quality in autistic children.

In conclusion, this study provides the first empirical evidence demonstrating that telehealth—a highly accessible expert-patient protocol—can effectively improve the well-being of autistic children. By interacting with experts through the digital platform, parents were able to create a supportive family environment, which resulted in significant improvements in their children's MVPA and sleep quality. This study establishes a novel treatment model that addresses the needs of families with autistic children and highlights the potential of telehealth in digital societies. We anticipate that this approach could have broad applications, extending beyond enhancing the quality of life for autistic individuals to potentially benefiting other populations in need of remote support and intervention.

## 5 Study limitations

This study, while offering important insights, acknowledges several limitations. The relatively small sample size may limit the generalizability of the findings, undersocring the need for larger and more diverse cohorts to enhance applicability across various socio-economic backgrounds and geographic locations. Furthermore, the lack of control over external influences, such as variations in family dynamics and school environments, may have affected the outcomes. Future research should consider effective strategies to account for these variables or adopt a more comprehensive analytical approach to better isolate the true effects of the intervention. Additionally, the focus on child outcomes did not sufficiently evaluate changes in parental behaviors and attitudes towards health promotion. Investigating how parental engagement and education through telehealth might influence their health behaviors could provide further insights into child outcomes. Lastly, while the study hypothesized that improvements in sleep quality might be linked to increased GABA levels and reduced anxiety, the underlying biological mechanisms remain largely unexplored. Future research should aim to clarify these physiological changes associated with telehealth interventions, including hormonal responses related to exercise.

## Data Availability

The raw data supporting the conclusions of this article will be made available by the authors, without undue reservation.

## References

[1] Zeidan J Fombonne E Scorah J Ibrahim A Durkin MS Saxena S Global prevalence of autism: a systematic review update. Autism Res. (2022) 15(5):778–90. 10.1002/aur.2696 35238171 PMC9310578

[2] Cortese S Wang F Angriman M Masi G Bruni O. Sleep disorders in children and adolescents with autism spectrum disorder: diagnosis, epidemiology, and management. CNS Drugs. (2020) 34(4):415–23. 10.1007/s40263-020-00710-y 32112261

[3] Andersen IM Kaczmarska J McGrew SG Malow BA. Melatonin for insomnia in children with autism spectrum disorders. J Child Neurol. (2008) 23(5):482–5. 10.1177/0883073807309783 18182647

[4] Doyen C Mighiu D Kaye K Colineaux C Beaumanoir C Mouraeff Y Melatonin in children with autistic spectrum disorders: recent and practical data. Eur Child Adolesc Psychiatry. (2011) 20(5):231–9. 10.1007/s00787-011-0162-8 21359552

[5] Barry L Holloway J McMahon J. A scoping review of the barriers and facilitators to the implementation of interventions in autism education. Res Autism Spectr Disord. (2020) 78:101617. 10.1016/j.rasd.2020.101617

[6] Liang X Haegele JA Tse AC-Y Li M Zhang H Zhao S The impact of the physical activity intervention on sleep in children and adolescents with autism spectrum disorder: a systematic review and meta-analysis. Sleep Med Rev. (2024) 74:101913. 10.1016/j.smrv.2024.101913 38442500

[7] Zhang Z Peng P Zhang D. Executive function in high-functioning autism spectrum disorder: a meta-analysis of fMRI studies. J Autism Dev Disord. (2020) 50(11):4022–38. 10.1007/s10803-020-04461-z 32200468

[8] Liang X Haegele JA Healy S Tse AC-Y Qiu H Zhao S Age-related differences in accelerometer-assessed physical activity and sleep parameters among children and adolescents with and without autism spectrum disorder: a meta-analysis. JAMA Netw Open. (2023) 6(10):e2336129. 10.1001/jamanetworkopen.2023.36129 37801316 PMC10559179

[9] Tse CYA Lee HP Chan KSK Edgar VB Wilkinson-Smith A Lai WHE. Examining the impact of physical activity on sleep quality and executive functions in children with autism spectrum disorder: a randomized controlled trial. Autism. (2019) 23(7):1699–710. 10.1177/1362361318823910 30663324

[10] Khor SPH McClure A Aldridge G Bei B Yap MBH. Modifiable parental factors in adolescent sleep: a systematic review and meta-analysis. Sleep Med Rev. (2021) 56:101408. 10.1016/j.smrv.2020.101408 33326915

[11] Gregory AM Sadeh A. Sleep, emotional and behavioral difficulties in children and adolescents. Sleep Med Rev. (2012) 16(2):129–36. 10.1016/j.smrv.2011.03.007 21676633

[12] Tuckson RV Edmunds M Hodgkins ML. Telehealth. N Engl J Med. (2017) 377(16):1585–92. 10.1056/NEJMsr1503323 29045204

[13] Gajarawala SN Pelkowski JN. Telehealth benefits and barriers. J Nurse Pract. (2021) 17(2):218–21. 10.1016/j.nurpra.2020.09.013 33106751 PMC7577680

[14] Chiang S-L Shen C-L Chen L-C Lo Y-P Lin C-H Lin C-H. Effectiveness of a home-based telehealth exercise training program for patients with cardiometabolic multimorbidity: a randomized controlled trial. J Cardiovasc Nurs. (2020) 35(5):491–501. 10.1097/JCN.0000000000000693 32511110

[15] Schopler E Reichler RJ Renner BR. The Childhood Autism Rating Scale (CARS). Los Angeles, CA: Western Psychological Services (2010).

[16] Morgenthaler T Alessi C Friedman L Owens J Kapur V Boehlecke B Practice parameters for the use of actigraphy in the assessment of sleep and sleep disorders: an update for 2007. Sleep. (2007) 30(4):519–29. 10.1093/sleep/30.4.519 17520797

[17] Quante M Kaplan ER Rueschman M Cailler M Buxton OM Redline S. Practical considerations in using accelerometers to assess physical activity, sedentary behavior, and sleep. Sleep Health. (2015) 1(4):275–84. 10.1016/j.sleh.2015.09.002 29073403

[18] Romeo DM Bruni O Brogna C Ferri R Galluccio C De Clemente V Application of the sleep disturbance scale for children (SDSC) in preschool age. Eur J Paediatr Neurol. (2013) 17(4):374–82. 10.1016/j.ejpn.2012.12.009 23352289

[19] Bruni O Ottaviano S Guidetti V Romoli M Innocenzi M Cortesi F The sleep disturbance scale for children (SDSC) construct ion and validation of an instrument to evaluate sleep disturbances in childhood and adolescence. J Sleep Res. (1996) 5(4):251–61. 10.1111/j.1365-2869.1996.00251.x 9065877

[20] Rostami Haji Abadi M Zheng Y Wharton T Dell C Vatanparast H Johnston J Children with autism spectrum disorder spent 30 min less daily time in moderate-to-vigorous physical activity than typically developing peers: a meta-analysis of cross-sectional data. Rev J Autism Dev Disord. (2023) 10(1):144–57. 10.1007/s40489-021-00262-x

[21] Ketcheson L Hauck J Ulrich D. The effects of an early motor skill intervention on motor skills, levels of physical activity, and socialization in young children with autism spectrum disorder: a pilot study. Autism. (2017) 21(4):481–92. 10.1177/1362361316650611 27354429

[22] Ledford JR Lane JD Shepley C Kroll SM. Using teacher-implemented playground interventions to increase engagement, social behaviors, and physical activity for young children with autism. Focus Autism Other Dev Disabil. (2016) 31(3):163–73. 10.1177/1088357614547892

[23] Obrusnikova I Cavalier AR. Perceived barriers and facilitators of participation in after-school physical activity by children with autism spectrum disorders. J Dev Phys Disabil. (2011) 23(3):195–211. 10.1007/s10882-010-9215-z

[24] Healy S Marchand G Williams E. “I’m not in this alone” the perspective of parents mediating a physical activity intervention for their children with autism spectrum disorder. Res Dev Disabil. (2018) 83:160–7. 10.1016/j.ridd.2018.08.014 30218986

[25] Bull FC Al-Ansari SS Biddle S Borodulin K Buman MP Cardon G World health organization 2020 guidelines on physical activity and sedentary behaviour. Br J Sports Med. (2020) 54(24):1451–62. 10.1136/bjsports-2020-102955 33239350 PMC7719906

[26] Paruthi S Brooks LJ D'Ambrosio C Hall WA Kotagal S Lloyd RM Consensus statement of the American academy of sleep medicine on the recommended amount of sleep for healthy children: methodology and discussion. J Clin Sleep Med. (2016) 12(11):1549–61. 10.5664/jcsm.6288 27707447 PMC5078711

[27] Kohyama J. Which is more important for health: sleep quantity or sleep quality? Children. (2021) 8(7):542. 10.3390/children8070542 34202755 PMC8304732

[28] Ruscio J. A probability-based measure of effect size: robustness to base rates and other factors. Psychol Methods. (2008) 13(1):19–30. 10.1037/1082-989X.13.1.19 18331151

[29] Nogueira HA de Castro CT da Silva DCG Pereira M. Melatonin for sleep disorders in people with autism: systematic review and meta-analysis. Prog Neuropsychopharmacol Biol Psychiatry. (2023) 123:110695. 10.1016/j.pnpbp.2022.110695 36584862

[30] Åslund L Arnberg F Kanstrup M Lekander M. Cognitive and behavioral interventions to improve sleep in school-age children and adolescents: a systematic review and meta-analysis. J Clin Sleep Med. (2018) 14(11):1937–47. 10.5664/jcsm.7498 30373682 PMC6223553

[31] Huang WY Ho RS-T Tremblay MS Wong SH-S. Relationships of physical activity and sedentary behaviour with the previous and subsequent nights’ sleep in children and youth: a systematic review and meta-analysis. J Sleep Res. (2021) 30(6):e13378. 10.1111/jsr.13378 34235808

[32] Tse AC Lee PH Zhang J Chan RC Ho AW Lai EW. Effects of exercise on sleep, melatonin level, and behavioral functioning in children with autism. Autism. (2022) 26(7):1712–22. 10.1177/13623613211062952 35083939

[33] Stone MR Stevens D Faulkner GEJ. Maintaining recommended sleep throughout the week is associated with increased physical activity in children. Prev Med. (2013) 56(2):112–7. 10.1016/j.ypmed.2012.11.015 23201000

[34] Covington LB Patterson F Hale LE Teti DM Cordova A Mayberry S The contributory role of the family context in early childhood sleep health: a systematic review. Sleep Health. (2021) 7(2):254–65. 10.1016/j.sleh.2020.11.010 33436342

[35] Vernhet C Dellapiazza F Blanc N Cousson-Gélie F Miot S Roeyers H Coping strategies of parents of children with autism spectrum disorder: a systematic review. Eur Child Adolesc Psychiatry. (2019) 28(6):747–58. 10.1007/s00787-018-1183-3 29915911

[36] Monteleone P Maj M Fusco M Orazzo C Kemali D. Physical exercise at night blunts the nocturnal increase of plasma melatonin levels in healthy humans. Life Sci. (1990) 47(22):1989–95. 10.1016/0024-3205(90)90432-Q 2273939

[37] Carlson LA Pobocik KM Lawrence MA Brazeau DA Koch AJ. Influence of exercise time of day on salivary melatonin responses. Int J Sport Physiol Perform. (2019) 14(3):351–3. 10.1123/ijspp.2018-0073 30160559

[38] Buman MP Phillips BA Youngstedt SD Kline CE Hirshkowitz M. Does nighttime exercise really disturb sleep? Results from the 2013 national sleep foundation sleep in America poll. Sleep Med. (2014) 15(7):755–61. 10.1016/j.sleep.2014.01.008 24933083

[39] Frimpong E Mograss M Zvionow T Dang-Vu TT. The effects of evening high-intensity exercise on sleep in healthy adults: a systematic review and meta-analysis. Sleep Med Rev. (2021) 60:101535. 10.1016/j.smrv.2021.101535 34416428

[40] Coxon JP Cash RFH Hendrikse JJ Rogasch NC Stavrinos E Suo C GABA concentration in sensorimotor cortex following high-intensity exercise and relationship to lactate levels. J Physiol. (2018) 596(4):691–702. 10.1113/JP274660 29159914 PMC5813602

[41] Maddock RJ Casazza GA Fernandez DH Maddock MI. Acute modulation of cortical glutamate and GABA content by physical activity. J Neurosci. (2016) 36(8):2449–57. 10.1523/JNEUROSCI.3455-15.2016 26911692 PMC6705493

[42] Kalueff AV Nutt DJ. Role of GABA in anxiety and depression. Depress Anxiety. (2007) 24(7):495–517. 10.1002/da.20262 17117412

